# The Innate Immune System and Inflammatory Priming: Potential Mechanistic Factors in Mood Disorders and Gulf War Illness

**DOI:** 10.3389/fpsyt.2020.00704

**Published:** 2020-07-23

**Authors:** Kyle J. Trageser, Maria Sebastian-Valverde, Sean X Naughton, Giulio Maria Pasinetti

**Affiliations:** ^1^ Department of Neurology, Mount Sinai School of Medicine, New York, NY, United States; ^2^ Geriatric Research, Education and Clinical Center, James J. Peters Veterans Affairs Medical Center, Bronx, NY, United States

**Keywords:** Gulf War Illness, mood disorders, neuroinflammation, microglia, therapeutics, innate immunity, cytokines, inflammation

## Abstract

Gulf War Illness is a chronic multisystem disorder affecting approximately a third of the Veterans of the Gulf War, manifesting with physical and mental health symptoms such as cognitive impairment, neurological abnormalities, and dysregulation of mood. Among the leading theories into the etiology of this multisystem disorder is environmental exposure to the various neurotoxins encountered in the Gulf Theatre, including organophosphates, nerve agents, pyridostigmine bromide, smoke from oil well fires, and depleted uranium. The relationship of toxin exposure and the pathogenesis of Gulf War Illness converges on the innate immune system: a nonspecific form of immunity ubiquitous in nature that acts to respond to both exogenous and endogenous insults. Activation of the innate immune system results in inflammation mediated by the release of cytokines. Cytokine mediated neuroinflammation has been demonstrated in a number of psychiatric conditions and may help explain the larger than expected population of Gulf War Veterans afflicted with a mood disorder. Several of the environmental toxins encountered by soldiers during the first Gulf War have been shown to cause upregulation of inflammatory mediators after chronic exposure, even at low levels. This act of inflammatory priming, by which repeated exposure to chronic subthreshold insults elicits robust responses, even after an extended period of latency, is integral in the connection of Gulf War Illness and comorbid mood disorders. Further developing the understanding of the relationship between environmental toxin exposure, innate immune activation, and pathogenesis of disease in the Gulf War Veterans population, may yield novel therapeutic targets, and a greater understanding of disease pathology and subsequently prevention.

## Introduction

**Figure 1:**
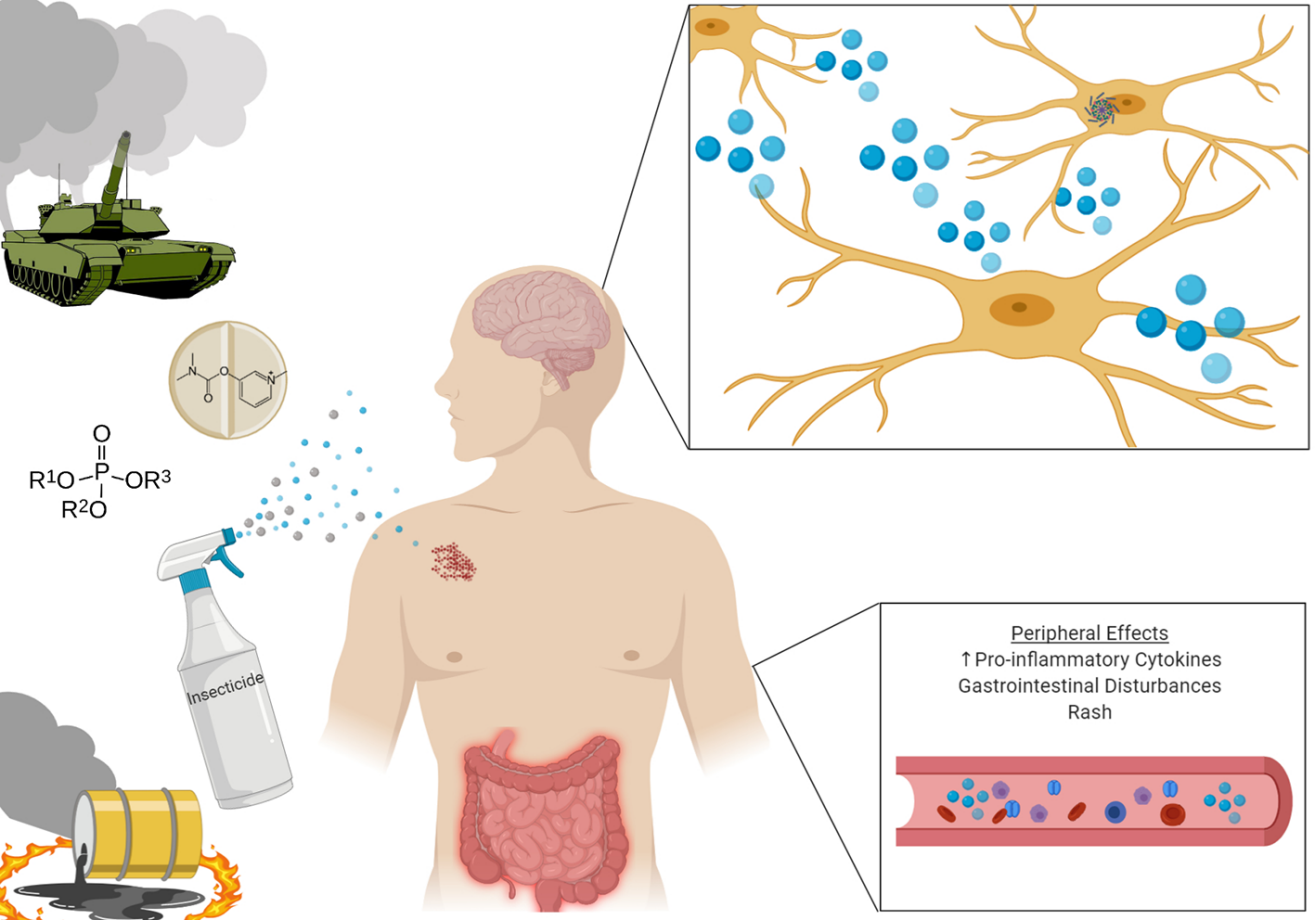
Veterans deployed to the Gulf Theatre were exposed to a wide array of neurotoxins, with potent neuroinflammatory effects. Upon return from deployment, many Veterans developed constellations of symptoms including rash, gastrointestintal disturbances, and cognitive impairment. Investigations have shown heightened inflammatory markers both peripherally and centrally in those diagnosed with Gulf War Illness.

Gulf War Illness (GWI) is a chronic multi-symptom illness affecting approximately one-third of the 700,000 U.S. troops deployed to the Persian Gulf region to combat the invasion of Kuwait in 1990–1991 (1). During the intervening conflict, deployed troops were exposed to a myriad of neurotoxins previously unseen during wartime. Soldiers were exposed to two classes of acetylcholinesterase inhibitors: organophosphates in the form of pesticides, and chemical weapons, as well as carbamates in the form of pesticides and the prophylactic anti-nerve agent treatment pyridostigmine bromide. Additionally, deployed troops were exposed to other insecticides and insect repellants such as permethrin, N, N-Diethyl-meta-toluamide (DEET), and lindane. Additional exposures to hazards include smoke from oil well fires and depleted uranium used in armor piercing rounds (2) (**Figure 1**).

Upon returning home, deployed Gulf War Veterans (GWV) started exhibiting constellations of symptoms including fatigue, gastrointestinal disturbances, dermatologic pathology (especially rashes), and cognitive impairment, which could not be reliably explained by the presence of other illnesses. As a result, large scale longitudinal studies were undertaken to investigate the relationship between those who served in the Persian Gulf and the development of these symptoms. These symptoms exhibit a high prevalence throughout all demographics of society, however when compared to non-deployed veterans a significant unexplained increase in the rates of these symptoms is noted in those deployed to the Gulf (3).

The prevalence of chronic multi symptom illness (CMI) is nearly double in deployed vs non-deployed GWV (28.9% vs 15.8%) (4). However, the occurrence of CMI in non-deployed veterans does raise interesting questions. CMI has now also been reported in veterans of the more recent wars in Iraq and Afghanistan, although to a lesser degree than in GWV. Rates of CMI are higher in deployed vs non-deployed veterans of the Iraq and Afghanistan wars (5). A potential explanation for this phenomenon is that physical and psychological stressors experienced during military training may stimulate CMI in certain predisposed individuals. Veterans of the Iraq and Afghanistan wars were also exposed to toxic smoke from burn pits on military bases (6). Thus, it is possible that stress from training or combat may act as a priming factor which later synergizes with inflammatory toxins (i.e. pesticides and nerve agents in Gulf War Veterans or toxic smoke from burn pits in later generation Veterans) to produce CMI.

Disruption of the innate immune system and inflammation has been correlated with GWI (7). Veterans presenting with the disease show alterations in brain structures and in the integrity of the blood-brain barrier mediated by the immune system. Indeed, brain function in GWI is identical to that found in other immune-related conditions (8) and consequently, GWI has been proposed to be studied as a neuroimmune disease (9). GWI animal models have validated the involvement of neuroinflammatory mechanisms in the pathology of the disease (10), including the over-reactivity of astrocytes and microglia. This increased activation of immune cells was also directly observed in the brain of veterans with GWI (11), while inflammatory biomarkers such as elevated levels of pro-inflammatory cytokines, IL-1β, INF-γ, or IL-6 (12, 13) have been found in the serum of GWI patients. Increased concentrations of inflammatory cytokines are also associated with different mood disorders, including bipolar and major depressive disorders (14, 15). Dysfunction of the innate immune system might be behind the depressive behavior observed in veterans with GWI.

To date, only incremental progress has been made towards the creation of a therapeutic for GWI; there is no FDA approved therapy. In 2008, following the publication of the findings of the Research Advisory Committee on Gulf War Veterans’ Illness, it was determined that the top priority in the next phase of GWI research should be the identification of a treatment (2). A putative strategy for developing novel therapeutics to treat GWI may leverage the many shared neuroinflammatory pathologies of mood disorders and GWI (9, 16). In this review, we discuss the relationship between the innate immune system, exposure to environmental toxins, and the resultant neuroinflammation leading to the development of Gulf War Illness. Furthermore, we propose that the pathomechanisms by which Gulf War Illness occurs possesses significant overlap with those found in many mood disorders, leading to a larger than expected concomitant rate of diagnosis in individuals with Gulf War Illness. Understanding this complex relationship may provide the opportunity to develop novel therapeutic strategies as well as a method to prevent similar disease.

## Innate Immunity

The immune system’s response to an insult can be broken down into two overarching arms: the specifically responding adaptive immune system and the nonspecific innate immune system. Adaptive immunity is slow; to mount a response against previously encountered antigens, specific B, and T cell colonies are activated and grow to defend the host. In contrast, innate immune responses rapidly act to combat threats (17). This rapidity is essential and enables the body to act *via* nonspecific action while a targeted response by the adaptive immune system is prepared. An integral way in which the innate immune system participates in cellular defense is through the activation of various receptors and sensors to activate caspase-1, and subsequently induce an inflammatory response mediated by pro-inflammatory cytokines, such as IL-1β or IL-18, among others (18). In the central nervous system, the main innate immune cells of the brain, microglia, participate in the nonspecific innate immune response, driving neuroinflammation (19). Microglial mediated neuroinflammation is of particular interest in the pathology of GWI and has been recently demonstrated with *in vivo* PET imaging in individuals diagnosed with GWI. Individuals diagnosed with GWI demonstrated a heightened signal of TSPO, a marker of microglial activation. TSPO signal was found to be significantly elevated in cortical regions including the precuneus, prefrontal cortex, and the primary motor and somatosensory cortices (11). This direct evidence of neuroinflammation in individuals diagnosed with GWI underlies the importance of research into the mechanisms by which GWI toxins activate the innate immune system, causing lasting neuroinflammation.

### Inflammasome Assembly and Activation in Response to GWI Toxins

**Figure 2:**
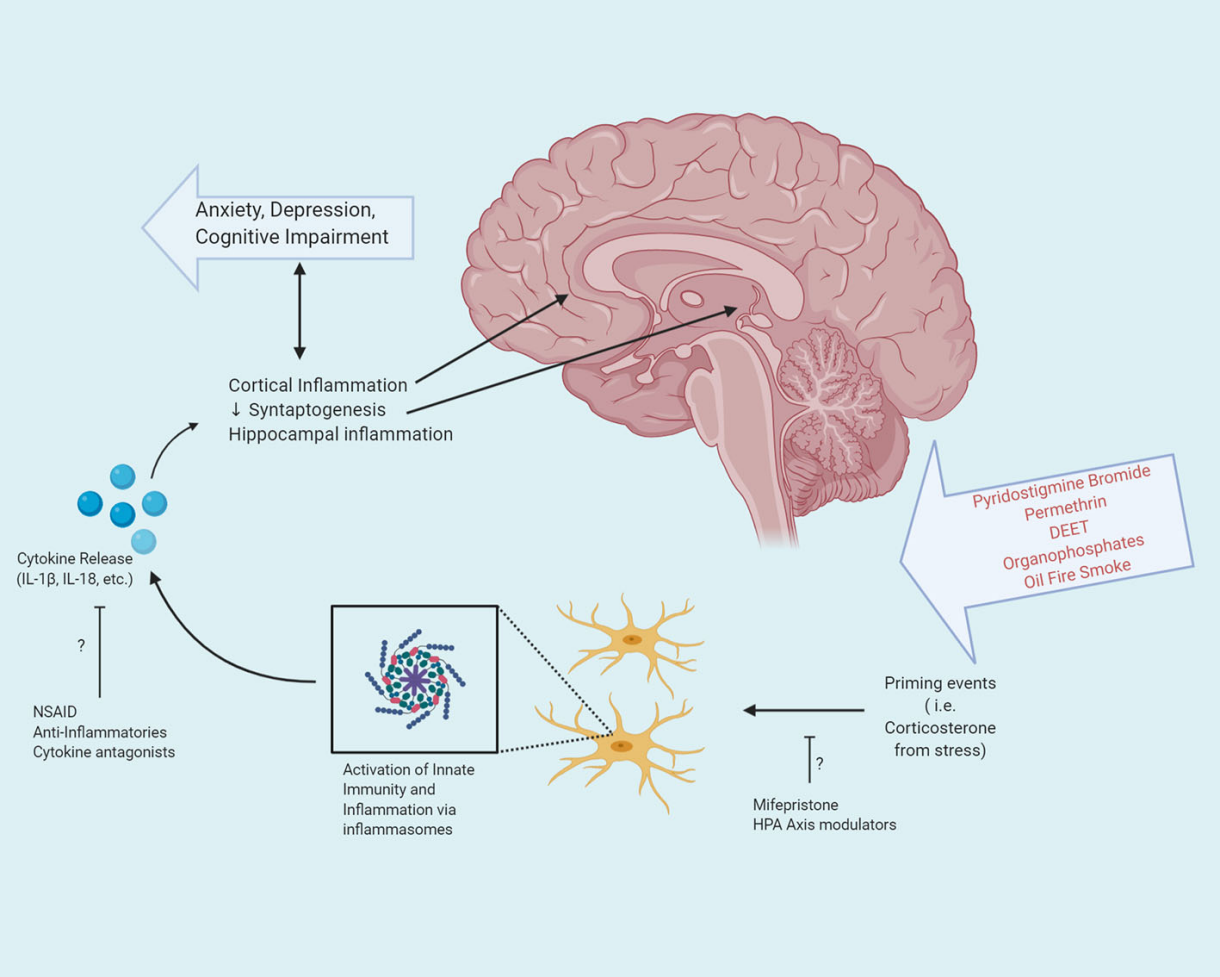
Exposure to the various neurotoxicants encountered during the Gulf War, in combination with prolonged stress can activate the innate immune system *via* inflammasomes. Activation of inflammasomes in microglia in the central nervous system results in the release of pro-inflammatory cytokines. These cytokines exert pleiotropic effects based upon their target destination and can result in both cortical and hippocampal inflammation, as well as decreased synaptogenesis, leading to anxiety, depression, and cognitive impairment. Potential therapeutic interventions may target the step of priming events or cytokine release, to attenuate or prevent the inflammatory cascade resulting in GWI.

Instrumental in the response of the innate immune system are inflammasomes: multiprotein complexes which assemble upon response to either an exogenous or endogenous insult. Components of the multiprotein complex include a pattern recognition receptor (PRR) protein, which may exist on the cell membrane or intracellularly, an adaptor protein, and an effector (20). One of the best characterized inflammasomes is the NOD-, LRR-, and pyrin domain containing protein 3 (NLRP3) complex, which plays an essential role in the initiation and propagation of the innate immune response. In the central nervous system, *Nlrp3* is highly expressed in microglia (21). The end result of activation of the NLRP3 inflammasome in microglia is the release of pro-inflammatory cytokines IL-1β and IL-18 (**Figure 2**).

The induction of the innate immune system by NLRP3 occurs in a two-step process, priming, and activation, in response to either an endogenous or exogenous insult. Externally derived threats are termed pathogen associated molecular patterns (PAMPs) and include bacterial endotoxins and glycoproteins. Those that originate from within the host are danger associated molecular patterns (DAMP) and include metabolites of purines, nucleic acids, reactive oxygen species, and heat shock proteins (22).

The ability of inflammasomes to respond to these DAMPs, often released during periods of extreme stress or toxicity, are a central tenant to the role of inflammasomes in the pathogenesis of GWI. DAMPs have been implicated in the development of neuroinflammation in both *in vitro* and *in vivo* animal models of GWI, with exposure of GWI associated chemicals leading to increases in various DAMPs, including the HMGB1 (23). The initial priming step is the result of the binding of a PAMP or DAMP to a PRR. The binding of the exogenous molecule lipopolysaccharide (LPS) or various endogenous molecules produced due to tissue injury such as high-mobility group box 1 protein (HMGB1) can result in the activation of the toll-like receptor 4 (TLR4)/NF-κB pathway. Under normal conditions, HMGB1 exists as a ubiquitous nuclear DNA binding protein, but during periods of cellular stress such as excitotoxicity, necrotic conditions, or traumatic brain injury, HMGB1 is released from neurons and astrocytes into the extracellular space and binds to the TLR complex of microglia, activating the transcription factor NF-κB. NF-κB activation leads to the transcription of zymogenic *pro-IL-1β* as well as the upregulation of *Nlrp3* (24, 25).

Following the priming step, the NLRP3 inflammasome may be activated, the induction of which can occur by various signals. A detailed mechanism for the activation step of inflammasome activity has not been elucidated in entirety, however various models have been put forward. Ion fluxes, such as those from K^+^, Ca^2+^, Na^+^, and Cl^-^) have been proposed to participate (26). For example, in the presence of damaged cells, a high concentration of extracellular ATP is often present. This high concentration induces the purine receptor P2X7 to open, allowing potassium to be released, the ionic effects of which are counteracted by calcium influx (27). The molecular mechanism by which these ion fluxes contribute to the release of pro-inflammatory cytokines are not fully known; however various *in vivo* models in which knock-out P2X7 mice were challenged with LPS, demonstrated significantly decreased secretion of IL-1β (28).

In response to activation, the innate immune cells rely on inflammatory cytokines from the TNF and Interleukin-1 family. A major participator in the inflammatory cascade is the pro-inflammatory cytokine, IL-1β. Once secreted, IL-1β exerts its inflammatory effects through binding to IL-1 receptors in the microenvironment and promoting the expression of cyclooxygenase-2 (*Cox-2*), phospholipase A2 (*Pla2*), and inducible nitric oxide synthase (*iNos*) of microglia and monocytes, each of which further participates in the inflammatory cascade. IL-1β also has the capability to increase the expression of adhesion molecules such as intercellular adhesion molecule-1 (ICAM1) on mesenchymal cells and vascular cell adhesion molecule-1 (VCAM1) on endothelial cells, functioning to increase adhesion and retention of immune cells to their target tissue (29).

In the brain, IL-1β exerts pleiotropic effects based upon cell type. In microglia and astrocytes, the binding of IL-1β to the IL-1 receptor leads to the activation of the NF-κB pathway. However, when bound to the IL-1 receptor of hippocampal neurons, rather than activating the NF-κB pathway, inflammatory levels of IL-1β activates the MAPK and CREB pathway thereby modulating synaptic strength and long-term potentiation. Interestingly, the effects of IL-1β in hippocampal neurons exhibits a dose-dependent response: low concentrations of IL-1β promote long-term potentiation and the formation of memories, whereas at elevated levels, a decrease in LTP and memory consolidation is noted (30). As memory deficits are disproportionately observed clinically in individuals with GWI, the effects of elevated levels of IL-1β in the hippocampus may be of particular interest in elucidating the pathophysiology of the disorder (31) (**Figure 2**). Neuroimaging in GWV with confirmed exposure to organophosphates reveals reduced volume of the CA2 and CA3/Dentate Gyrus subfields of the hippocampus (32, 33). Furthermore, elevated levels of IL-1β have been demonstrated *in vivo* in the hippocampus of animals in models of GWI (34). The association of IL-1β concentrations in hippocampal neurons may putatively serve as a target for therapeutic intervention.

### Sensitization of the Innate Immune System

The magnitude of inflammatory proteins released by microglia is plastic; rather than an all or nothing release, microglia are able to dynamically modulate their release of cytokines. Previous exposure of microglia to an insult can either increase or decrease the sensitivity and subsequent reactivity *via* the release of proinflammatory cytokines upon secondary exposure to an event (35). Demonstrated *in vivo*, when given an initial subthreshold dose of LPS, mice manifest regular, non-increased levels of IL-1β in the CNS. However, when a second dose of LPS is administered 24 h after the initial, elevated levels of IL-1β, TNF-α, and IL-12 were demonstrated in the CNS, despite an attenuation of the production of cytokines by the cells of the periphery (36). This sensitization provides valuable insight into the pathogenesis of various diseases, in which repeated exposure to subthreshold stressors may ultimately elicit a full, or even heightened response.

Subthreshold levels of cytoplasmic NLRP3 protein prevent the catalytic reaction of pro-interleukins from occurring. In chronic diseases with inflammatory components, upregulation of *Nlrp3* mRNA has been demonstrated including chronic periodontitis and diabetes mellitus (37). Mutations in the NLRP3 gene possess clinical relevancy in humans; when occurring in the capsase-1 domain of NLRP3, affected individuals exhibit uncontrolled excretion of IL-1β and IL-18 as part of the autoimmune disorder Muckle-Wells Syndrome, presenting with arthralgia, fatigue, and dermatological pathology (38).

Corticosterone, a glucocorticoid synthesized and secreted by the adrenal cortex during prolonged periods of stress, has been proposed to play a contributory role in the priming step of the innate immune system (39). Corticosterone has been shown to increase the basal level of mRNA expression of several proteins found in the NLRP3 inflammasome complex (40). This effect has been demonstrated both *in vitro* and *in vivo*; in primary macrophages derived from both human and mice, the treatment of cells with glucocorticoids was found to rapidly induce expression of *Nlrp3*. Furthermore, when these cells were assayed for IL-1β secretion, it was demonstrated that the administration of glucocorticoids greatly increased levels of mature IL-1β released (41). To investigate the effects of glucocorticoids on inflammasome priming *in vivo*, studies have been conducted administering methamphetamine, a potent mediator of neuroinflammation. Mice chronically pretreated with corticosterone, in a paradigm designed to mimic the physiological effects of prolonged stress, and subsequently administered methamphetamine, demonstrated significant increases in TNF-α, CCL-2, LIF, and IL-1β compared to animals given a single pretreatment dose of corticosterone prior to methamphetamine treatment (42). This exaggerated immune response induced by the chronic administration of corticosterone prior to the inflammatory insult also coincided with physiological abnormalities such as substantially increased dopaminergic cell death and increased numbers of activated microglia.

### GW Toxins and Innate Immunity

The neuroinflammation observed in individuals with GWI has been recapitulated both *in vivo* and *in vitro* as a result of the toxic exposure to the various chemicals encountered by deployed GWV. Interestingly, models have also investigated the contributory effects of sustained levels of physiological stress, which may serve a role in the sustained activation of microglia. Below we review various animal models of GWI and their key findings as they relate to the pathogenesis of neuroinflammation and systemic inflammation (**Figure 1**).

Permethrin (PER) is a type I pyrethroid insecticide which acts on voltage gated Na+ channels in neurons to produce a long chain of action potentials following a single stimulus (43). This insecticide is one of the many environmental exposures experienced by veterans of the first gulf war (2). Permethrin has been shown to act on voltage gated sodium channels present on microglia and stimulate the release of TNF-α in a dose and time dependent manner (44).

Numerous *in vivo* studies have reported increases in inflammatory markers after chronic exposure to permethrin when co-exposed with various combinations of PER, pyridostigmine bromide (PB), N, N-Diethyl-meta-toluamide (DEET), and/or chronic stress. Carreras and colleagues demonstrated that chronic exposure to PER, PB, and DEET, along with 5 min of restraint stress for 28 days produced a significant increase in the number of activated microglia in CA1-3 regions of the hippocampus in mice at time point of 3 months post-exposure. Interestingly, these findings were accompanied by increased anxiety-like behavior in an elevated plus maze behavioral test (45).

Chronic 10-day administration of PB and PER in the absence of chronic stress has been shown to increase inflammation, as detected by increases in the phosphorylation of NF-κB and STAT3 and corresponding increases in IL-1β, IL-6, and IFN-γ in the brains of mice 11 months post-exposure. IL-1β and IFN-γ were also elevated in the plasma taken from these mice. Animals in this study were also evaluated for fatigue and anxiety behaviors. At 3 months post-exposure the animals showed an increase in the amount of time spent immobile in a forced swim test, a finding suggested to mirror increases in fatigue and depression commonly observed in GWI patients. Additionally, the mice in this study were found to display disinhibition behavior as measured by an increased amount of time in the open arms of an elevated plus maze (46). While the reason for differences in elevated plus maze behavior between the studies by Carreras and colleagues and Joshi et al. has not been explicitly studied, it is likely a result of differences in the experimental protocols (i.e. use of chronic restraint stress and DEET).

An *in vivo* model of GWI demonstrated the ability of the toxins encountered during the Gulf War, pyridostigmine bromide and permethrin, with subsequent administration of corticosterone to stimulate stressful conditions, to activate the NLRP3 inflammasome in enteric glial cells. Rodents treated with pyridostigmine bromide and permethrin followed by corticosterone exhibited activation of TLR4 receptors and an upregulation in expression of *Nox2*- a producer of super-oxide, ultimately leading to the activation of the NLRP3 inflammasome, as well as upregulation of mRNA for *Nlrp3*, *Caspase-1*, and *Il-1β* (47).

While multiple animal models of GWI have been used, neuroinflammation remains a prominent feature across various combinations of Gulf War toxins and stressors. A recent study compared two models of GWI, PB+PER (Model 1) and PB+DEET+CORT+DFP (Model 2). Animals in Model 1 received treatment with PB and PER concurrently for 10 days; Model 2 animals received treatment with PB and DEET for 14 days, with administration of CORT on days 8–14 and a single dose of DFP on day 15. The authors showed increased expression of IL-1β, CCL-2, *Casp1*, and a trending increase in *Tnf-*α in the ventral hippocampus of mice in both models, with stronger increases seen in Model 2. Interestingly, *Hmgb1* showed a trending increase (34).

Research regarding the gut-brain-axis has received growing attention and excitement in recent years and may represent a potential mechanistic explanation for GWI. Seth and colleagues demonstrated that oral administration of PER+PB was associated with dysregulation of both the gut virome and bacteriome in mice leading to a weakening of tight junctions in the GI, as well as systemic inflammation, neuroinflammation and decreased BDNF expression in the frontal cortex. Interestingly, these mice displayed increased levels of the pro-inflammatory cytokine IL-6 in the frontal cortex. IL-6 levels in PER+PB treated mice could be reduced by treatment with antibiotic and antiviral compounds, a finding that suggests gut dysbiosis and bacteria-virus communication could be factors in neuroinflammation observed in GWI models (48).

O’ Callaghan and colleagues demonstrated that exposure to the sarin surrogate Diisopropyl fluorophosphate (DFP) produces neuroinflammatory effects, as measured by increases in several cytokines across multiple brain regions. Interestingly, increases in TNF-α, IL-1β, and OSM were further enhanced by prior administration of CORT to mimic physiological stress. Administration of PB and DEET did not enhance DFP-induced neuroinflammation, or produce neuroinflammation outright (49). A subsequent study compared the ability of various cholinesterase inhibitors to stimulate neuroinflammation with or without the prior administration of CORT. The study compared two organophosphate irreversible AchE inhibitors and two carbamate reversible AchE inhibitors. The organophosphates used in the study were DFP and Chlorpirifos-Oxon (CPO). Chlorpirifos-Oxon is the active metabolite of Chlorpirifos, an insecticide used in the first gulf war. The carbamates used in the study were PB and physostigmine (PHY). Physostigmine is a carbamate similar to PB, but with the ability to penetrate the blood-brain-barrier. Both DFP and CPO stimulated a robust increase in the levels of several inflammatory cytokines in the cortex and hippocampus of mice with corresponding activation of the downstream neuroinflammatory signaling effector STAT3, in a manner which could be exacerbated by prior treatment with CORT. The same neuroinflammatory effect was not observed with PB or PHY (50). A follow-up study looking at exposure to DFP, CPO or PHY alone or in combination with CORT found elevations (or lack thereof) of cytokines in various brain regions did not readily correlate with region specific changes in Ach from the various treatments. These results were interpreted to suggest that off target (non-AchE) actions of OPs may be chiefly responsible for the observed changes in neuroinflammation (51). An additional study from the same group demonstrated that the observed neuroinflammatory effects of DFP+CORT in mice extend to rats as well and are accompanied by changes in microdiffusivity as detected by MRI (52). A recent study using RNAseq analysis found 32 genes that were differentially expressed in both the cortex and hippocampus of mice treated using the same DFP+CORT exposure regime mentioned above. In particular these changes in gene expression were associated with functions such as cytokine-cytokine receptor interactions, regulation of chemokine production, and regulation of I-κB kinase/NF-κB signalling (53). Finally, it has also been observed that the neuroinflammatory changes resulting from GWI toxin exposures are not mirrored in the periphery. For example DFP alone elevated mRNA expression of several proinflammatory cytokines in the liver as had previously been reported in the brain (49), but chronic CORT exposure suppressed these changes in contrast to what had previously been observed in the brain. A similar pattern of DFP induced inflammation suppressed by CORT was also observed in the serum. The study also looked at PB+DEET exposure and found this treatment had no or reduced effect on mRNA expression of cytokines in the liver. Exposure to CORT with or without PB+DEET was associated with reductions in serum cytokine expression. Exposure to PB+DEET was associated with no or reduced expression of most proinflammatory cytokines in the serum. It should be noted however, that PB+DEET exposure did increase concentrations of IL-1α, IL-6, and IL-2 in the serum at some of the time points evaluated (2 and 72 h, 12 h, and 6 and 12 h, respectively) (10).

Autoimmunity may also play a role in the pathogenesis of GWI. A recent study examined the contributions of adaptive immune response in a PER + PB model of GWI. The PER metabolite 3-PBA was shown to haptenate albumin, and autoantibodies against 3-PBA-albumin were detected in the plasma of mice chronically exposed to PER+PB, as well as GWI patients and farm workers exposed to pyrethroid insecticides. 3-PBA-albumin was also shown to activate CD4+ T-helper and antigen responsive B-cells in ex vivo murine blood. These immune cells were also shown to be elevated in PER+PB exposed mice as well as GWI patients. Interestingly, the authors also reported an increase in infiltrating monocytes in the brains of mice chronically exposed to PER+PB. Additional evidence for a potential autoimmune component of GWI is supported by the detection of antibodies against a number of CNS proteins in GWI veterans as well as agricultural workers exposed to organophosphates. In particular autoantibodies against GFAP, Tau, MAP2, and myelin basic protein (MBP) were detected (54, 55).

Further interplay of the innate and adaptive arms of the immune systems have been hypothesized to contribute to the pathogenesis of GWI. Bridging the innate and adaptive immune systems is the process of antigen presentation, by various cells, including the macrophages of the innate immune system (56). In the process of antigen presentation, macrophages upregulate major histocompatibility complex class II (MHC Class II) to present to CD4^+^ T cells (57). Human leukocyte antigen (HLA) Class I, II, and III genes are transcribed from the MHC region of human chromosome 6. Dysfunction in HLA Class II genes have previously been demonstrated in Veterans with GWI and hypothesized to confer susceptibility to the myriad of environmental insults and exposures encountered during deployment. Interestingly, GWV deployed during this time with higher allelic frequency of HLA genes may provide protection from the development of GWI (58).

## Relationship Between Inflammation, Innate Immunity and Mood Disorders

Among the constellation of symptoms and illnesses present in GWI is a high rate of diagnosis of a concomitant mood disorder; interestingly, those with a comorbid mood disorder diagnosis exhibit increased severity of symptoms (59). In fact, among the multi-pronged diagnostic criteria as outlined by the Kansas definition of GWI, is cognition/mood impairments, fatigue, pain, respiratory dysfunction, and GI or dermatologic symptoms (60).

Various mood and psychiatric disorders were found to occur at a higher than expected level in deployed GWV when compared to non-deployed GWV. In addition, meta-analysis of 14 studies of GWV determined that there was greater than twice the odds ratio of developing depression compared to military personnel non-deployed during the Gulf War (61).

Considering this degree of concomitant diagnosis and many shared neuroinflammatory pathologies, we describe below a number of key neuroinflammatory findings of various neuropsychiatric disorders which may explain this concurrence.

One dilemma that must be addressed is the direction of causality of inflammation in psychiatric disorders; is inflammation the cause, or does it solely represent a symptom of underlying features of neurological dysfunction such as synaptic malformation, improper neurite outgrowth, or neurotransmitter dysfunction? The first shred of evidence about the relationship between pro-inflammatory cytokines and mood disorders was reported in 1991 by Smith (62). In this study, the administration of monokines to volunteers increased the rate of depressive disorders, which agreed with the higher prevalence of depression in patients with inflammatory conditions, such as rheumatoid arthritis. From then, different studies have shown that inflammation not only coincides with psychiatric disorders but that it also exacerbates the symptom severity in several syndromes. The impact of pro-inflammatory cytokines has been mostly studied in major depression disorders (MDD). In a meta-analysis of studies conducted by Dowlati *et al*., cytokine levels in patients with MDD were analyzed, and it was concluded that depression coincides with alterations of the inflammatory response system (63). Nevertheless, enhanced cytokine levels are also found in other psychological conditions, such as bipolar disorder (BPD) (64, 65), anorexia nervosa (66), panic and posttraumatic stress disorders (67), schizophrenia (68), and even neurodegenerative conditions including Alzheimer’s disease (69). When analyzing the causative relationship between cytokines alterations and psychiatric illnesses, a retrospective study of 3 million medical records in the Netherlands concluded that a history of hospitalization for infection was associated with an increased risk of later developing a mood disorder, including depression and bipolar disorder (70). Interestingly, chronic inflammation also seems to promote addictive behaviors. In a study that analyzed the genomic DNA isolated from 60 opioid-dependent, 99 alcohol-dependent patients, and 60 healthy nondependent controls, demonstrated that single nucleotide polymorphisms in the IL-1β gene was related to alcohol and opioids-dependency (71).

Shifting the focus toward the concentration of specific inflammatory proteins in neuropsychiatric patients, clinical studies found a significant augmentation in the concentration of IL-18 in patients with MDD panic disorder (72), and increased levels of IL-1β in depression and bipolar disorders (73), while TNF-α seems to mediate the production of anorexigenic peptides in anorexia nervosa (74). A meta-analysis about MDD published in 2012, which included 29 studies of the proinflammatory cytokines in the serum of 1548 patients––822 MDD, 726 healthy controls—confirmed that soluble IL-2 receptor, IL-6, and TNF-α levels are increased in MDD (trait markers), while, IL-1β, IL-2, IL-4, IL-8, and IL-10, are not statistically different from controls (63). A recent review summarizes alterations in serum cytokines in BPD; TNF-α, IL-6, and IL-8 are elevated during manic and depressive phases, whereas IL-2, IL-4, and IL-6 are increased during mania (75). Two more studies reported that IL-1β and IL-1 receptor serum levels of MDD, BPD, and schizophrenia patients are not statistically different from those of healthy controls (14), although tissue studies revealed increased levels of IL-1β and IL-1 receptor in the frontal cortex of BPD patients (76).

The relationship between mood disorders and inflammation has also been extensively studied in the laboratory, using both *ex vivo *cultures from depressed patients and animal models. For instance, olfactory bulbectomized rats present neuroendocrine, behavioral, and immune modifications similar to those found in MDD patients, which might be related to alterations of the inflammation-HPA axis, and inflammation-nerve growth factor-memory pathway (77). Several works using rat and mouse models of stress have demonstrated the influence of inflammatory cytokines in stress-induced depressive cases. Thus, mice subjected to chronic-mild stress present different symptoms of depression, including adrenocortical activation, decreased neurogenesis and behavioral alterations, as a consequence of elevated IL-1 levels in the brain (78) (For putative illustration of neuroimmune interactions and GWI pathogenesis see **Figure 2**). Acute immobilization stress-induced in rats increased levels of IL-1β and TNF-α, which correlated with depressive behaviors and impaired neurogenesis (79). Another animal model that explores the interaction between the immune system and mood alterations is administration of bacterial lipopolysaccharide (LPS). In rodents, intraperitoneal LPS injection promotes altered behaviors related to anxiety and depression, such as reduced social behavior, anhedonia, or decreased libido (78, 80, 81). These changes are associated with augmented concentrations of IL-1 (α and β), IL-6, and TNF-α (82). Furthermore, peripheral blood mononuclear cells (PBMC) cultures from schizophrenic patients produced higher concentrations of IL-18 and IL-1β, both spontaneously and upon stimulation with LPS (83).

## Targeting Inflammation in Mood Disorders and GWI: A Common Mechanism

Targeting the process of priming in neuroinflammation may yield novel treatment strategies to both treat, as well as prevent the pathogenesis of diseases with a chronic inflammation component, such as GWI or various neuropsychiatric disorders. As many psychiatric disorders display a number of commonalities with regards to neuroinflammation and GWI, there exists a potential area of overlap for which treatments may be effective. Described below are a selection of therapeutics targeting inflammation that have been trialed either in Gulf War Illness or in disorders shown to have similar neuroinflammatory effects.

Neuronal inflammation frequently coincides with psychiatric disorders such as MDD, BPD, or schizophrenia. Thus, the most obvious question that arises is whether anti-inflammatory drugs are useful for the treatment of these diseases. Several studies attempted to treat psychiatric disorders with different types of approved anti-inflammatory drugs; however, there are important inconsistencies about the effectiveness of the studied drugs. Four major categories of anti-inflammatory drugs have been typically evaluated; non-steroidal anti-inflammatory drugs (NSAIDs), polyunsaturated fatty acids (PUFAs), and cytokines inhibitors, and the antibiotic minocycline (84–86). NSAIDs are a group of anti-inflammatory drugs that inhibit cyclooxygenase (COX) enzymes, blocking the synthesis of prostaglandins. There are several NSAIDs available with different commercial names and specificity for COX-1 and COX-2. PUFAs are a family of long-chain n‐3 polyunsaturated fatty acids that exert anti-inflammatory effects by reducing the expression of inflammatory genes, probably through targeting the NF-κB pathway (87). Cytokine inhibitors include nonpeptidic molecules that suppress cytokine synthesis, soluble receptors that sequestrate the synthesized cytokines, and autoantibodies that neutralize complement-mediated cell death (88). Among this latest group, anti-TNF-α drugs deserve particular attention. Finally, minocycline is a second-generation tetracycline antibiotic that presents anti-inflammatory properties independent from its anti-bacterial effect.

In 2014, Köhler et al. published a meta-analysis based on 14 studies from randomized clinical trials, and 6262 patients with depressive symptoms, or MDD. They concluded that the treatment with anti-inflammatory drugs (specifically, both cyclooxygenase-2 inhibitors and anti-cytokine therapies) reduced depressive symptoms. However, there was considerable heterogeneity among the conclusions reached by the studies included in the meta-analysis (89). Another extensive qualitative review evaluated the effectiveness of the four types of anti-inflammatory drugs as add-on therapies for BPD, MDD, and schizophrenia (85). This study inferred that PUFAs improved symptoms of MDD patients, although the beneficial effects depended on the eicosapentaenoic acid content of their diet (90). There was no evidence of improvement in schizophrenic patients treated with PUFAs (91), while there were mixed results for subjects with bipolar disorders (92). Regarding the use of NSAID, the cyclooxygenase-2 inhibitor effectively improved the symptomatology of depressed patients, having a more modest impact on schizophrenia and no effect at all on BPD. The anti-TNFα drug Infliximab has been tested in MDD patients resistant to other anti-depressants. In general, the drug did not improve the symptoms of the disease, and only those patients with a high baseline of inflammatory biomarkers (>5 g/l of the high-sensitivity C-reactive protein), showed some improvement. In this subgroup of individuals, Infliximab administration ameliorated anhedonia, anxiety, depressed mood, and suicidal thoughts (93). No anti-TNFα therapies have been tested in BPD and schizophrenic patients. There are limited data regarding the use of minocycline to treat psychiatric disorders; however, the administration of the antibiotic as an add-on to the conventional therapies improved the symptoms of MDD and schizophrenic patients (94, 95).

Antidepressant medications have been additionally investigated for their anti-inflammatory capacities. Hannestad *et al.* in a meta-analysis published in 2011 in Nature Neuropsychopharmacology, analyzed the effect of antidepressant drugs in the serum levels of inflammatory cytokines, including TNFα, IL-1β, and IL-6 (96). Patients diagnosed with MDD and treated with approved medicines for depression presented lower serum levels of IL-1β than before the treatment. However, the reduction of the IL-6 concentration was less clear, and TNF-α levels remained unaltered. Interestingly, when subgrouping the data according to the class of antidepressant, serotonin reuptake inhibitors significantly reduced IL-6 and TNF-α levels, while other antidepressants did not alter these proinflammatory markers (96). Partially contrasting with this study, another meta-analysis published in 2018, concluded that the use of antidepressant for the treatment of MDD reduced IL-4, IL-6, and IL-10, while the drugs did not significantly change IL-2, TNF-α, IFN-γ, or CRP. According to this study, IL-1β was only reduced by serotonin reuptake inhibitors, whereas other types of antidepressants did not affect this cytokine (97). Nevertheless, both studies agreed about the higher levels of inflammatory cytokines found in MDD patients, which could contribute to depressive symptoms.

The heterogeneous response found among individuals evinces the necessity to characterize the inflammatory profiles of psychiatric patients to provide personalized and more effective therapy.

## Therapeutic Strategies in Gulf War Illness

There is currently no standard FDA approved therapy used for the treatment of GWI. In fact, the Research Advisory Committee on Gulf War Veterans’ Illnesses has designated the identification of effective treatments for GWI as the highest priority in GWI research (2). ClincialTrials.gov currently lists 52 different clinical studies examining a number of different therapeutic interventions for GWI. However, very few of the listed studies have successfully reached completion and published their results. Several studies involving non-pharmacological interventions (i.e. acupuncture, cognitive behavioral therapy) and nutritional supplementation studies have been performed in patients with GWI (98). For the purposes of the present review the focus will remain solely on nutritional and pharmacological agents tested in rodent models as well as symptomatic GWV.

### Coenzyme Q10

Coenzyme Q10 (CoQ10) is concentrated mainly in the mitochondria and is integral in energy production (99). Recent evidence also suggests that CoQ10 can also serve as an antioxidant by protecting the cell membrane from reactive oxygen species (ROS). As ROS have been hypothesized to play a contributing role in the pathogenesis of GWI, as well as numerous psychiatric conditions, CoQ10 has been trialed as a treatment for GWI (100, 101). In randomized, double-blind, placebo-controlled study of individuals diagnosed with GWI, participants were administered CoQ10 at either 100 mg a day, 300 mg a day, or as a placebo for 3.5 months. Individuals treated with 100 mg of CoQ10 daily reported significant improvements in General Self-Reported Health compared to baseline; however this effect was limited only to male participants. In an objective measure of physical function, improvements in individuals in the 100 mg group were significantly increased compared to placebo, with the effect present in both male and female participants (102).

### Mifepristone

A randomized double-blind cross-over trial was conducted in Gulf War Veterans with diagnosed chronic multi-symptom illness using the type II glucocorticoid receptor antagonist mifepristone. Subjects received 200 mg/day for 6 weeks and were assessed across a battery of cognitive tests. Treatment was associated with significant improvements in verbal learning. However, mifepristone was not associated with improvements in working memory, visual learning, or on the overall composite score. Treatment was also associated with increased levels of plasma cortisol and ACTH, as would be expected from chronic GR antagonist administration (103).

### Doxycycline

One of the less common theories surrounding GWI is that underlying systemic infection with Mycoplasma may be a causal factor in the disorder. Based on this hypothesis a randomized, double-blind, placebo-controlled clinical trial was conducted in GWI patients with detectable Mycoplasma DNA in their blood. The treatment used in the study was 200mg per day of Doxycycline over a period of 12 months. The study failed to find any improvement in the primary outcome measure of physical health function after 12 months. Additionally the study found no significant differences in pain, fatigue, cognitive symptoms, and mental health following 12 months treatment (104).

### Potential Future Directions

Various medications currently FDA approved may hold potential in being trialed in cases of GWI. Suramin, a medication previously used in the treatment of African Trypanosomiasis, can act a non-selective inhibitor of purinergic signaling and may have efficacy in attenuating the activation step of NLRP3 activity by blocking the P2x7 receptor. Interestingly, Suramin has recently been trialed in Phase I/II, low dosages as a treatment for Autism Spectrum Disorders to determine safety. While the study was unable to draw robust statistical conclusions regarding the efficacy of Suramin as a treatment, modest improvements in ASD symptomology were present in individual in the experimental group (105). As purines serve as a DAMP for inflammasome activation, the use of Suramin may be of interest as a potential treatment in GWI.

Anakinra, an interleukin-1 receptor antagonist (IL-1Ra) is currently FDA approved for the treatment of rheumatoid arthritis (106). In addition to the currently approved indication, Anakinra has been trialed in a number of other conditions with inflammatory components, including stroke and ALS, although there have been varying degrees of success regarding changes in clinical outcomes and changes in serum levels of inflammatory cytokines (107). Although varying efficacy has been demonstrated, the safety profile, dearth of approved GWI treatments, and heightened levels of IL-1β found in GWI may warrant further trials.

### Rodent Studies

Joshi and colleagues demonstrated that the Peroxisome proliferator-activated receptor alpha (PPAR-α) agonist Oleoylethanolamide (OEA) was sufficient to reverse cognitive deficits in the Barnes maze in a mouse model of GWI (PB+PER). OEA treatment was also able to reverse PB+PER induced depressive and disinhibition behaviors as measured *via* forced swim test and elevated plus maze. OEA treatment was also found to effectively reverse PB+PER stimulated increases in the phosphorylation of the NF-κB subunit p65, as well as STAT3 phosphorylation. OEA was also effective in reversing PB+PER stimulated increases in the levels of IL-1β, IL-6, and IFN-γ (46). OEA is an endogenous acylethanolamide with known anti-inflammatory actions. The exact mechanism of anti-inflammatory action by OEA is currently unknown however several mechanisms have been proposed such as the alterations in inflammatory signaling pathways and genes down stream of various receptors known to be targeted by acylethanolamides such as CB receptors, PPARs, and TRPVs (108).

The antibiotic minocycline has anti-inflammatory actions and has been shown to be effective in preventing CORT+DFP induced increases in inflammatory cytokine expression. Co-treatment of minocycline with CORT+DFP was associated with decreases in the expression of TNF-α, CCL2, IL-1β, LIF, and OSM in the frontal cortex and hippocampus of treated mice (49). Minocycline is a tetracycline antibiotic which has been shown to be neuroprotective, immunomodulatory, and anti-inflammatory in a number of different animal models. Some of the proposed mechanisms for these additional effects include inhibition of iNOS, MMPs, and PLA_2_. As well as inhibition of caspases-1 and -3 and inhibition of PARP-1 (109).

A study by Seth and colleagues (see above section *GW toxins and innate immunity*) tested co-exposure with antibiotics (Neomycin + Enrofloxacin) or the antiviral compound Ribavirn in conjunction with PB+PER. The study showed that treatment with Ribavirn resulted in a gut viral composition that was similar to control mice that were not exposed to PB+Per, a result not found with the antibiotic treatment. Antibiotic treatment was associated with preventing changes in gut bacteria diversity that occurred with PB+PER exposure, whereas antiviral treatment was not. Interestingly, both antibiotic as well as antiviral therapy were associated with prevention of increases in serum IFNγ and IL6 after exposure to PB+PER (48). While antibiotic therapy has previously been tested in symptomatic Gulf War Veterans without success, the strategy of deliberately altering gut microbiome composition has not yet been tested in GWI patients.

In addition to pharmaceuticals, natural phytochemicals have also been trialed *in vivo* as treatments for the neuroinflammation present in GWI. One such natural supplement, curcumin, has been demonstrated to have potent anti-inflammatory and anti-oxidant effects *via* its action through a number of pathways, including the NF-κB and STAT3 signaling paths (110). At 9 weeks of age, male rats were exposed daily to DEET, permethrin, and pyridostigmine bromide, plus physical restraint for a period of 28 days. Animals were subsequently administered curcumin daily or a vehicle injection. Animals exposed to GW toxins and treated with curcumin exhibited improved cognitive and mood function, hippocampal neurogenesis, and reduced hippocampal inflammation, when compared to vehicle treated, GW toxin exposed (111). Demonstrated *in vivo* efficacy of curcumin to attenuate neuroinflammation sequelae of GWI suggests further preclinical and clinical trials are warranted.

## Conclusion

With the high incidence of GWI in deployed GWV and a current lack of approved treatment, there exists a need for new trials targeting novel mechanisms. Neuroinflammation as catalyzed by the innate immune system represents a major contributor to the pathogenesis of GWI, along with the concurrence of concomitant mood disorders. Future therapeutic strategies that leverage the commonalities in these pathomechanisms may yield promising developments in creating a treatment. Furthermore, GWI has a high degree of overlap with the fields of environmental toxicology, psychiatry, immunology, and neurology; an interdisciplinary effort across multiple fields is essential for a better understanding of its underlying mechanism. Interdisciplinary research may also provide important contributions to each of these respective fields. The development of therapeutics for the treatment of GWI may potentially be repurposed for treating other disorders, highlighting the continued value in pursuing both mechanistic and therapeutic driven studies.

## Author Contributions

All authors contributed to the conceptualization, writing, and editing of this manuscript.

## Funding

This study was supported by grant number P50 AT008661-01 from the NCCIH and ODS. We acknowledge that the contents of this review do not represent the views of the NCCIH, the ODS, the NIH, or the United States Government.

## Conflict of Interest

The authors declare that the research was conducted in the absence of any commercial or financial relationships that could be construed as a potential conflict of interest.
